# Randomized trial to assess the potential role of ascorbic acid and statin for post-contrast acute kidney injury prevention

**DOI:** 10.1007/s11255-023-03806-8

**Published:** 2023-09-24

**Authors:** Abdelwahab Hashem, Mahmoud Laymon, Mostafa Elgamal, Mohammed Hegazy, A. M. Elmeniar, Huda Refaie, Yasser Osman

**Affiliations:** 1 Urology Department, Urology and Nephrology Center, Mansoura, Egypt; 2 Urology Department, 30th June Urology and Nephrology Centre, Ismailia, Egypt; 3 Urology Department, Shebin Elkom Teaching Hospital, Menofia, Egypt; 4 Urology Department, Met-Ghamr Urology and Nephrology Hospital, Dakahlia, Egypt; 5 Radiology Department, Urology and Nephrology Center, Mansoura, Egypt

**Keywords:** Acute kidney injury (AKI), Ascorbic acid, Computed tomography, Contrast, Nephropathy, Statin

## Abstract

**Purpose:**

To evaluate the effect of using statins and ascorbic acid for the prevention of post-contrast acute kidney injury (PC-AKI) in patients undergoing urologic diagnostic elective contrast-enhanced computed tomography (CECT).

**Methods:**

This registered trial (NCT03391830) was for statin naïve patients underwent elective CECT. Patients were randomized allocated to two groups: the first group received atorvastatin 80-mg the day before the study and atorvastatin 40-mg two hours before the CECT and for continue on atorvastatin 40-mg two days after CECT; plus ascorbic acid 500 mg with atorvastatin. The other group received two tablets of placebo once/daily before the procedure and for another 3 days. The primary outcome was to assess the incidence PC-AKI.

**Results:**

The baseline parameters were comparable between both groups. The final median (interquartile range “IQR”) serum creatinine were 0.80 (0.60, 1.00) and 0.80 (0.60, 1.00), respectively, with insignificant *p*-value (*p* = 0.8). The median (IQR) final estimated GFR were 95.2 (72.8, 108.1) and 88.6 (71.9, 111.0) mL/min in placebo and statin plus ascorbic acid groups, respectively (*p* = 0.48). The eGFR difference median (IQR) were − 6.46 (− 11.72, − 4.18) and − 6.57 (− 13.38, − 3.82) ml/min in placebo and statin plus ascorbic acid groups, respectively (*p* = 0.58). PC-AKI occurred in 11 patients (9.8%) in placebo group and in 3 patients (3%) in statin plus ascorbic acid group (*p* = 0.04).

**Conclusions:**

Statin and ascorbic acid did not statistically improve neither serum creatinine nor eGFR values in patient underwent CECT. However, it can decrease the incidence of the clinically insignificant PC-AKI.

## Introduction

Iodinated contrast materials are commonly used in the urology daily practice. It can be used for intravenous contrasted imaging studies ranging from intravenous pyelogram, contrast enhanced computed tomography (CECT) up to diagnostic and intervention angiography. However, the iodinated contrast has been involved in many cases of kidney injury [1].

The definition, incidence, and risks of post-contrast acute kidney injury (PC-AKI) have not been clearly defined [1]. PC-AKI has been defined by the European Society of Urogenital Radiology (ESUR) as an increase in serum creatinine within 3 days after intravascular contrast medium (CM) administration by > 25% or 0.5 mg/dl, compared to its baseline value, without an alternate etiology [2]. The Kidney Disease: Improving Global Outcomes (KDIGO) criteria are now being assumed as the standard for PC-AKI, defined as an increase serum creatinine increase of ≥ 1.5–1.9 times or in serum creatinine of ≥ 0.3 mg/dl, compared to its baseline value [3].

The Contrast Media Safety Committee of ESUR highly recommend volume expansion with plenty of intravenous fluid over oral hydration [4]. Oral hydration was comparable and effective with the intravenous hydration in two randomized trials [5, 6].

Several medications have been advised to obviate PC-AKI. Some of them showed conflicting results regarding PC-AKI prevention such as; calcium channel blockers, mannitol, dopamine, atrial natriuretic peptide, L-arginine, selective D1 receptor partial agonist known as fenoldopam, prostaglandin E1, loop diuretic as furosemide, and endothelin receptor antagonist [2]. Novel anti-ischemic agents “trimetazidine and nicorandil”, *N*-acetylcysteine, ascorbic acid, and statin, in a recent meta-analysis, showed some sort of a protection in PC-AKI [7].

For patients undergoing computed tomography (CT) with a nonionic, low-osmolality contrast agent, *N*-acetylcysteine plus volume expansion using intravenous fluid could prevent PC-AKI in patients with chronic renal insufficiency [8]. However, oral *N*-acetylcysteine has mild gastrointestinal symptoms as nausea and vomiting that occurred in 23% of patients[9].

A lot of studies try to investigate medications for prevention of PC-AKI in cardiac and vascular patients. On the other hand, sparse of them investigate those medications in the field of urology [10]. The screening and prevention of PC-AKI had apparently wide variation in daily practice with some practice conflict with the literature recommendations and so further researches and areas in need of improved practice guidelines might have great opportunities [11].

In this trial, we will try to investigate the combined use of both ascorbic acid and statin for patients undergoing urologic diagnostic contrast enhanced CT and their effect of kidney injury caused by CECT.

## Patients and methods

This 4-day, randomized, placebo-controlled, parallel-group, registered clinical trial (ClinicalTrials.gov identifier NCT03391830) was conducted between January 2018 and January 2020 at tertiary urology center outpatients clinic for patients with serum creatinine ≤ 1.4 mg/dl, underwent elective contrast-enhanced computed tomography (CECT). The study protocol was approved by Institutional Review Boards (R/17.06.05). Fully informed consent was obtained from all patients, in line with Good Clinical Practice and the Declaration of Helsinki.

Eligible patients were statin naïve patients, or not on statin treatment for at two weeks planned for CECT. Patients with history of liver disease, rhabdomyolysis, contrast hypersensitivity, and multiple myeloma were excluded. None of the patients received metformin, nonsteroidal anti-inflammatory drugs, theophylline, *N*-acetylcysteine or iodinated contrast within the last two weeks before enrollment in the study.

Patients were randomized allocated to two groups: the first group received Lipitor (atorvastatin—Pfizer Egypt, under Authority of: Pfizer INC., USA) 80-mg the day before the study and atorvastatin 40-mg two hours before the CECT and for continue on atorvastatin 40-mg two days after CECT; plus C-Retard 500 mg (ascorbic acid—Hikma Pharma, October City, Egypt) with atorvastatin. The other group received two tablets of placebo once/daily before the procedure and for another 3 days.

The primary outcome was to assess the incidence of PC-AKI in both groups, defined as The KDIGO criteria [3]. The secondary outcome was to detect the difference in serum creatinine within 72 h after administration of contrast agent. The difference is calculated by subtracting serum creatinine value 72 h after contrast administration from baseline serum creatinine value. Estimated Glomerular Filtration Rate (eGFR) was calculated from serum creatinine values using the 4-variable equation of Modification of Diet in Renal Disease (MDRD) [12].

Normal saline (0.9 percent) was given intravenously at a rate of 1 ml per kilogram of body weight per hour for 12 h before and 12 h after administration of the contrast agent. All patients were encouraged to drink if they were thirsty. The dose of nonionic, low-osmolality CM (Iopromide; Ultravist-300, Schering, Berlin, Germany) was 75 ml for all patients. Serum creatinine was measured immediately before administration of the CM, and 73 h after, and one week in case of increase serum creatinine post CECT.

A per-protocol analysis is performed and includes all randomized patients who are compliant to treatment and complete their follow up. The null hypothesis assumed there is no difference between ascorbic acid and statin arm and placebo on the effect on PC-AKI.

The power of the study was calculated using the G*Power program (University of Düsseldorf, Düsseldorf, Germany) to determine an adequate sample size based on previous trial assuming a maximum serum creatinine differences in the statin and placebo groups [13]. Using the priori test with accuracy mode calculation and an effect size convention of 0.37 for Wilcoxon–Mann–Whitney test, with α error probability of 0.05, provided 85% power with expected 10% dropout rate for a total sample size of 244 patients.

Statistical analysis was performed using the IBM Statistical Package for the Social Sciences (SPSS®), version 22.0 for Windows (IBM Corp., Armonk, NY, USA). Qualitative data are described as numbers (percentages). Quantitative data are described as median (interquartile range). For the normally distributed variables, the independent sample *t* test was used for comparison between groups. The comparison of the mean scores in each group at different time intervals was carried out using the paired sample *t* test. Chi-square tests were used to compare the categorical data of both groups. A *p* ≤ 0.05 was considered to be statistically significant.

## Results

**Fig. 1:**
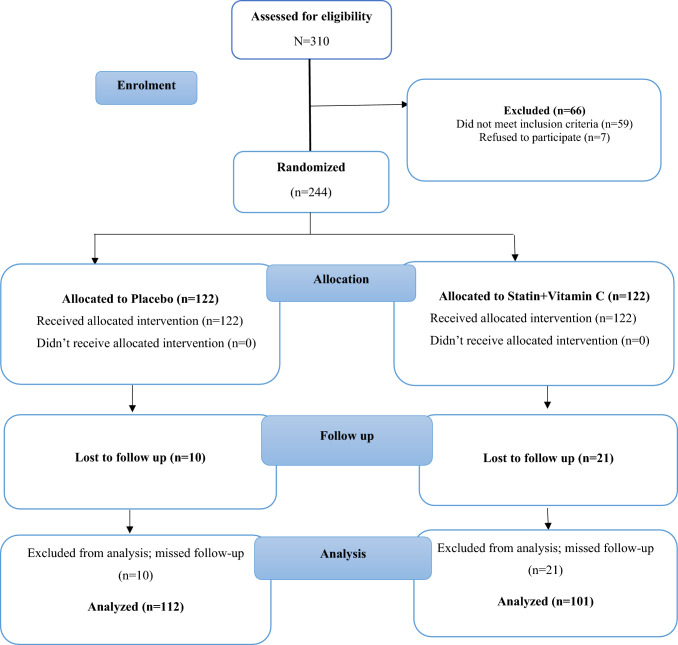
CONSORT flowchart

A total of 310 were assessed for eligibility and 244 patients meet our inclusion criteria. Of the 244 patients randomized, 31 patients lost follow-up visits and are excluded from final analysis as shown in CONSORT flowchart (Fig. 1). Patient characteristics of the two groups did not differ significantly (Table 1).

**Table 1:** Baseline patients’ characteristics

	Placebo, *N* = 112	Statin plus Ascorbic acid, *N* = 101	*p*-value
Age mean(SD)	48 (38.5, 59.5)	51 (43, 61)	0.08
Gender *N* (%)			
Male	50 (44.6)	48 (47.5)	
Female	62 (55.4)	53 (52.5)	0.67
BMI mean (SD)	32.0 (26.6, 36.0)	32.3 (27.8, 36.5)	0.20
Hypertension (HTN) *N* (%)	26 (23.2)	34 (33.7)	0.09
HTN duration median (range)	2.5 (2, 3)	3 (2, 4)	0.19
DM *N* (%)	14 (12.5)	21 (20.8)	0.10
DM duration median (range)	4.5 (1.5, 7.5)	2 (1.5, 4)	0.27
Neuropathy *N* (%)	7 (6.3)	7 (6.9)	0.84
Retinopathy *N* (%)	3 (2.7)	6 (5.9)	0.24

The baseline median (interquartile range “IQR”) serum creatinine was 0.80 (0.60, 0.98) mg/dl in placebo group and 0.80 (0.60, 1.00) mg/dl in statin plus ascorbic acid group with insignificant *p*-value (*p* = 0.93). Using the 4-variable equation of Modification of Diet in Renal Disease (MDRD), the median (IQR) baseline estimated GFR were higher in placebo group, 97.1 (80.7, 114.9) mL/min than in statin plus ascorbic acid groups, 94.6 (78.2, 120.7) mL/min and the *p*-value, as well, did not reach the statistical significance (*p* = 0.66).

The final median (interquartile range “IQR”) serum creatinine were 0.80 (0.60, 1.00) mg/dl in placebo group and 0.80 (0.60, 1.00) mg/dl in statin plus ascorbic acid group, with insignificant *p*-value (*p* = 0.8). The median (IQR) final estimated GFR were 95.2 (72.8, 108.1) mL/min in placebo group and 88.6 (71.9, 111.0) mL/min in statin plus ascorbic acid group, with insignificant *p*-value (*p* = 0.48) (Table 2).

**Table 2:** Baseline and final patients renal profile characteristics

	Placebo, *N* = 112	Statin plus ascorbic acid, *N* = 101	*p*-value
Contrast medium-induced nephropathy *N* (%)	11 (9.8)	3 (3)	**0.04**
Baseline serum creatinine, mg/dl Median (IQR)	0.80 (0.60, 0.98)	0.80 (0.60, 1.00)	0.93
Final serum creatinine, mg/dl Median (IQR)	0.80 (0.60, 1.00)	0.80 (0.60, 1.00)	0.80
Baseline estimated GFR, mL/min Median (IQR)	97.1 (80.7, 114.9)	94.6 (78.2, 120.7)	0.66
Final estimated GFR, mL/min Median (IQR)	95.2 (72.8, 108.1)	88.6 (71.9, 111.0)	0.48
The estimated GFR difference, mL/min median (IQR)	− 6.46 (− 11.72, − 4.18)	− 6.57 (− 13.38, − 3.82)	0.58

Bold indicates statistical significance

The estimated GFR difference median (IQR) were − 6.46 (− 11.72, − 4.18) and − 6.57 (− 13.38, − 3.82) ml/min in placebo and statin plus ascorbic acid groups, respectively, with insignificant *p*-value (*p* = 0.58).

**Fig. 2:**
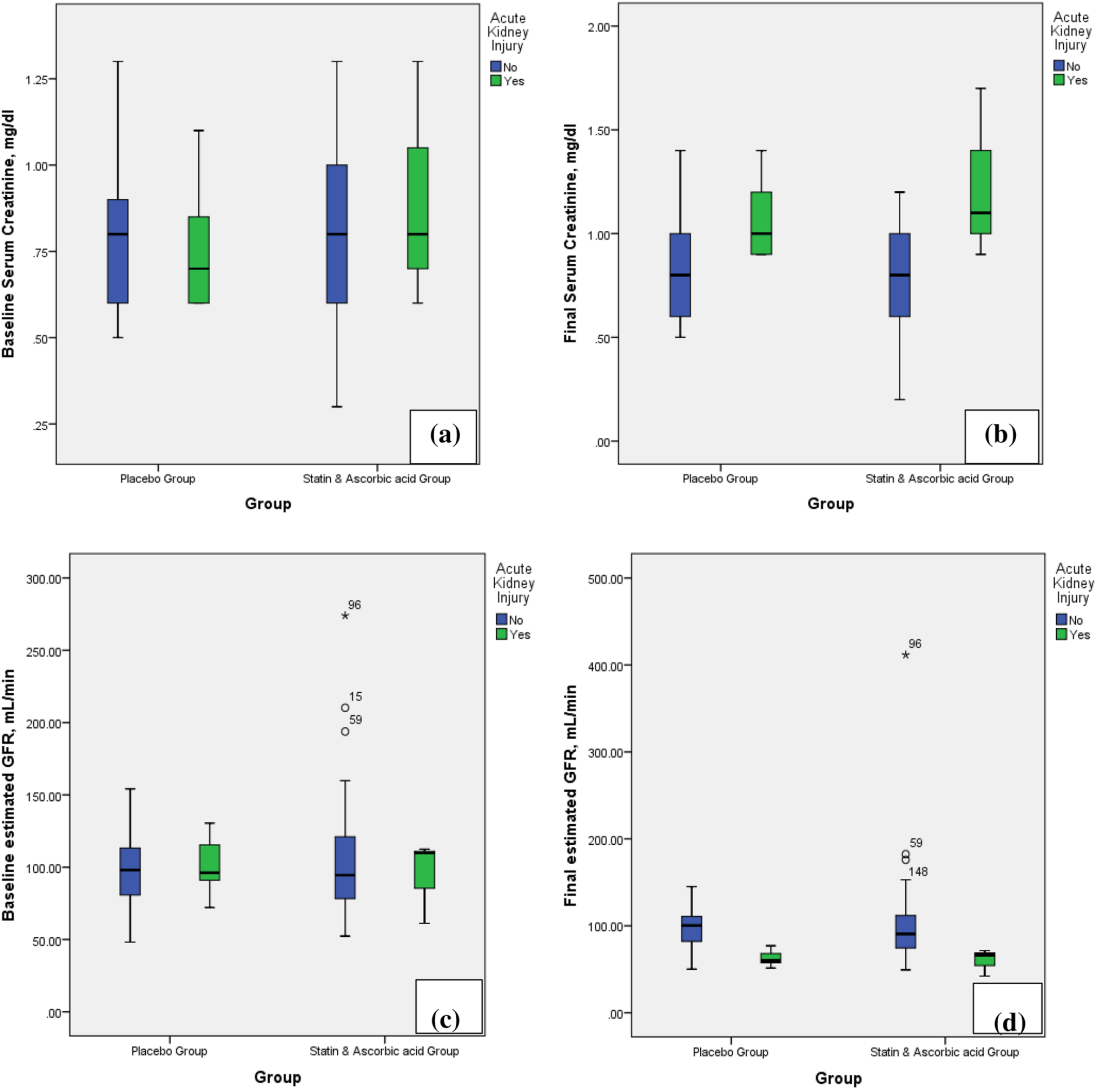
Boxplot graph for the comparison between patients with and without acute kidney injury, regarding baseline (**a**) and final (**b**) serum creatinine and baseline (**c**) and final (**d**) estimated glomerular filtration rate (GFR)

Regarding the contrast medium-induced nephropathy, defined in this trial by increase serum creatinine by 0.3 mg/dl, it occurred in 11 patients (9.8%) in placebo group and in 3 patients (3%) in statin plus ascorbic acid group, with statistical significant *p*-value (*p* = 0.04) (Table 2). The creatinine and estimated GFR difference between patients with and without acute kidney injury are shown in Fig. 2. All these patients were followed up by serial serum creatinine and all returned to their normal values.

Compared to the patients baseline values, in the placebo group, the mean ± SD serum creatinine rise from 0.80 ± 0.22 0.82 ± 0.24 mg/dl (*p* = 0.11). However, the mean ± SD estimated GFR decreased significantly from 100.45 ± 24.60 to 92.85 ± 24.78 ml/min (*p* < 0.001).

The same occurred in the statin plus ascorbic acid group, compared to the its baseline values, the mean ± SD serum creatinine rise from 0.80 ± 0.21 to 0.81 ± 0.23 mg/dl (*p* = 0.14). The mean ± SD estimated GFR decreased significantly from 102.27 ± 34.47 to 96.17 ± 24.31 ml/min (*p* = 0.003) (Table 3).

**Table 3:** Final patients renal profile characteristics, compared to their baseline profile

	Baseline Serum Creatinine, mg/dl Mean ± SD	Final Serum Creatinine, mg/dl Mean ± SD	*p*-value	Baseline estimated GFR, mL/min Mean ± SD	Final estimated GFR, mL/min Mean ± SD	*p*-value
Placebo, *N* = 112	0.80 ± 0.22	0.82 ± 0.24	0.11	100.45 ± 24.60	92.85 ± 24.78	** < 0.001**
Statin plus ascorbic acid, *N* = 101	0.80 ± 0.21	0.81 ± 0.23	0.14	102.27 ± 34.47	96.17 ± 24.31	**0.003**

Bold indicates statistical significance

## Discussion

Post-contrast acute kidney injury (PC-AKI) should be defined as ≥ 1.5–1.9 times baseline increase in serum creatinine or increase in serum creatinine of ≥ 0.3mg/dl (The KDIGO definition of AKI) in the 48–72 h following CM administration  [3]. Two large meta-analyses of 19,000 patients who had received IV CM showed PC-AKI incidences of 5.0–6.4% [3].

Published literature about post-contrast acute kidney injury (PC-AKI) mainly focuses on cardiac intervention as intravascular coronary angiography and rarely focuses on patients undergoing urological elective contrast-enhanced computed tomography. In a comparative study, PC-AKI was noted in 8.3% and 29.8% in urology and cardiology patients, respectively [10]. In Weisbord et al. trial, patients with eGFRs < 60 ml/min underwent non-emergent CECT in the inpatient and outpatient, about 3.5% of them manifested an increase in serum creatinine ≥ 0.5 mg/dl [14].

Although the pathogenesis of PC-AKI is not fully understood, Contrast agents have direct cytotoxic effects on vascular endothelial cells, or renal tubular epithelial cells resulting in inflammation, necrosis, and apoptosis [15]. The official guidelines published by the ESUR and the American College of Radiology and both recommend prophylactic hydration (1.0–1.5 mL/kg/h) in patients at risk for AKI at least 6 h before and after CM administration [2].

Atorvastatin (statin) reduces the PC-AKI inflammatory response and apoptosis of renal tubular epithelial cells. Treatment with atorvastatin also decreases the serum expression levels of Interleukins-1b, I Interleukins-6, Interleukins-8 and Tumor necrosis factor alpha. Atorvastatin might ameliorate PC-AKI through anti-apoptosis pathway associated with the Bcl-2/caspase-3 [16].

Oxidative stress shares some role in the pathogenesis of PC-AKI. Intracellular ROS are produced when the nephrons are exposed to contrast medium. When intracellular ROS synthesis rate overcomes the rate of their excretion, leads to oxidative-antioxidant imbalance, causing lipid peroxidation of biofilm, intracellular protein degeneration, and DNA damage [17].

Vitamin C, an antioxidant, attenuates the oxidative damage caused by CM and might effectively prevent PC-AKI. It has been reported that vitamin C effectively countered oxidative damage by reducing and scavenging reactive oxygen species that damage macromolecules such as lipids, proteins, and DNA [18, 19].

The PRATO-ACS trial showed that the incidence of PC-AKI was significantly lower in the statin group (6.7%) than in controls (15.1%) with significant *p* = 0.003. Vitamin C plus saline administration is effective to reduce the risk of CI-AKI by 25% [20]. In our trial, PC-AKI occurred in 11 patients (9.8%) in placebo group and in 3 patients (3%) statin plus ascorbic acid group (*p* = 0.04).

In Tepel et al., RCT, prophylactic oral *N*-acetylcysteine for CKD patient with GFR < 50 ml/minute who underwent elective contrast-enhanced computed tomography prevents the reduction in renal function. In the control and *N*-acetylcysteine group, the mean serum creatinine concentration increased from 2.4 ± 1.3 to 2.6 ± 1.5 mg/dl (*p* = 0.18) and decrease from 2.5 ± 1.3 to 2.1 ± 1.3 mg/dl (*p* < 0.001), respectively [8].

In our RCT, the mean ± SD serum creatinine rise in both groups, from 0.80 ± 0.22 mg/dl to 0.82 ± 0.24 mg/dl (*p* = 0.11) and from 0.80 ± 0.21 mg/dl to 0.81 ± 0.23 mg/dl (*p* = 0.14), in placebo and statin plus ascorbic acid groups, respectively.

Also, the mean ± SD estimated GFR decreased significantly in both groups with better significance in the statin plus ascorbic acid group (*p* < 0.001) versus (*p* = 0.003). In placebo group, mean ± SD estimated GFR decreased significantly from 100.45 ± 24.60 to 92.85 ± 24.78 ml/min (*p* < 0.001). The same occurred in the statin plus ascorbic acid group, the mean ± SD estimated GFR decreased significantly from 102.27 ± 34.47 to 96.17 ± 24.31 ml/min (*p* = 0.003). The difference may be attributed to difference in baseline characteristics, our patients were normal serum creatinine, however, in Tepel et al. the patients were CKD patients.

We found that PC-AKI occurred in 9.8% and 3% in placebo and statin plus ascorbic acid group, respectively, according to new KDIGO criteria (serum creatinine ≥ 0.3 mg/dl), while using pre-procedure intravenous fluid. In Weibord study, 3.5% demonstrated a rise in serum creatinine ≥ 0.5 mg/dl. Although only 6% of outpatients received pre- and post-procedure intravenous fluid [14]. We think that may be attributed the difference in PC-AKI definition.

Our trial has some limitations, we exclude patients with serum creatinine > 1.4 mg/dl. We choose serum creatinine as it is the most commonly used laboratory value as a screening test before CECT [11]. The incidence of PC-AKI increases dramatically after a threshold serum creatinine of 1.2–1.5 mg/dl is reached. So, randomised trials with large patient numbers for this category are warranted. Also, we calculate GFR using MDRD in patients without apparent basal renal disease, not the renal scintigraphy.

## Conclusion

The use of statin plus ascorbic acid to prevent the post-contrast acute kidney injury did not statistically improve neither serum creatinine nor estimated glomerular filtration rate values. However, statin plus ascorbic acid can decrease the incidence of the clinically insignificant post-contrast acute kidney injury, compared to the placebo.

## Data Availability

Data is available after approval of ethical committee.
